# Diprosopus: A Rare Case of Craniofacial Duplication and a Systematic Review of the Literature

**DOI:** 10.3390/genes14091745

**Published:** 2023-08-31

**Authors:** Viola Trevisani, Eleonora Balestri, Manuela Napoli, Stefano Giuseppe Caraffi, Maria Chiara Baroni, Francesca Peluso, Anna Colonna, Lorenzo Iughetti, Giancarlo Gargano, Andrea Superti-Furga, Livia Garavelli

**Affiliations:** 1 Medical Genetics Unit, Department of Mother and Child, Azienda USL-IRCCS di Reggio Emilia, 42123 Reggio Emilia, Italyfrancesca.peluso@ausl.re.it (F.P.); 2 Post-Graduate School of Pediatrics, Department of Medical and Surgical Sciences of the Mother, Children and Adults, University of Modena and Reggio Emilia, 41125 Modena, Italy; 3 Neonatal Intensive Care Unit, Department of Mother and Child, Azienda USL-IRCCS di Reggio Emilia, 42123 Reggio Emilia, Italy; 4 Neuroradiology Unit, Department of Diagnostic Imaging and Laboratory Medicine, Azienda USL-IRCCS di Reggio Emilia, 42123 Reggio Emilia, Italy; 5 Department of Medical and Surgical Science, Alma Mater Studiorum University of Bologna, 40126 Bologna, Italy; 6 Department of Biomedical Technologies, School of Dentistry, University of Siena, 53100 Siena, Italy; 7 Division of Genetic Medicine, Lausanne University Hospital (CHUV), University of Lausanne, 1011 Lausanne, Switzerland

**Keywords:** diprosopus, diprosopia, craniofacial duplication, case report

## Abstract

In 1990, Gorlin et al. described four types of craniofacial duplications: (1) single mouth with duplication of the maxillary arch; (2) supernumerary mouth laterally placed with rudimentary segments; (3) single mouth with replication of the mandibular segments; and (4) true facial duplication, namely diprosopus. We describe a newborn born with wide-spaced eyes, a very broad nose, and two separate mouths. Workup revealed the absence of the corpus callosum and the presence of a brain midline lipoma, wide sutures, and a Chiari I malformation with cerebellar herniation. We conducted a systematic review of the literature and compared all the cases described as diprosopus. In 96% of these, the central nervous system is affected, with anencephaly being the most commonly associated abnormality. Other associated anomalies include cardiac malformations (86%), cleft palate (63%), diaphragmatic hernia (13%), and disorder of sex development (DSD) (13%). Although the facial features are those that first strike the eye, the almost obligate presence of cerebral malformations suggests a disruptive event in the cephalic pole of the forming embryo. No major monogenic contribution has been recognized today for this type of malformation.

## 1. Introduction

The name “craniofacial duplication” is used to describe a wide number of anomalies that range from less severe forms of embryo clefting of the face to diprosopus. Approximately 40 cases have been reported, but only 29 reports contain enough data to be analyzed. In 1982, Barr et al. [1] categorized facial duplication into four distinct types based on the absence or presence of duplication of the eyes, nose, pituitary gland, maxilla, and mandible, but it was the late Bob Gorlin who provided the most recent classification of this condition.

In 1990, Gorlin et al. [2] highlighted four types of craniofacial duplications: (1) single mouth with duplication of the maxillary arch, (2) supernumerary mouth laterally placed with rudimentary segments, (3) single mouth with replication of the mandibular segments, and (4) true facial duplication, namely diprosopus. Various authors suggested that duplication of the pituitary gland could be the least severe form of this anomaly [3,4]. Craniofacial duplication can happen together with a broad variety of anomalies, in particular those affecting the central nervous system, among which anencephaly is the most severe malformation described in diprosopus [5,6,7,8,9,10,11,12,13,14,15]. 

It is a rare condition with an approximate incidence of 1 case in 180,000–15,000,000 births [10].

The causes and pathogenesis related to this condition are currently unknown, but the possible role of sonic hedgehog (Shh) receptors on embryonic-level control of early craniofacial tissue differentiation emerges in the literature. Unfortunately, very limited knowledge exists about the relationship between the disruption of the Shh pathway and the phenotypic outcome in the craniofacial region, and many questions still need to be answered with future studies [16].

Prognosis is typically poor, leading to death within the first months of life for cardiorespiratory arrest, but most of the cases described were stillbirths or fetuses aborted due to natural or voluntary causes after the performance of prenatal ultrasounds.

In minor craniofacial duplications, maxillofacial surgery was performed for mouth repositioning and closure of accessory mouths. Cases of diprosopus were instead hardly treated and brought to surgical correction; this condition would theoretically require multiple interventions anyway.

## 2. Materials and Methods

### 2.1. Case Report

For the description of the clinical case, we followed the CARE 13 guidelines, whose checklist will be found in the Supplementary Materials (File S1) [17].

### 2.2. Systematic Review

The literature research was conducted independently by 2 investigators (LG and VT) using the PubMed CENTRAL databases. The aim of the review is to analyze the state-of-the-art of the condition of the clinical case described: diprosopus.

The following combinations of keywords were used: diprosopus OR diprosopia OR craniofacial duplication.

The research was limited to papers published in English from May 1970 to March 2023, using the filter “Human”. The reference lists of retrieved studies have also been reviewed to identify studies that may have not been spotted by the search strategy.

#### 2.2.1. Inclusion Criteria

Papers included in our analysis were required to meet all of the following criteria: reports of patients with diprosopus and reports focused primarily on the clinical features of diprosopus. 

#### 2.2.2. Data Extraction

**Figure 1:**
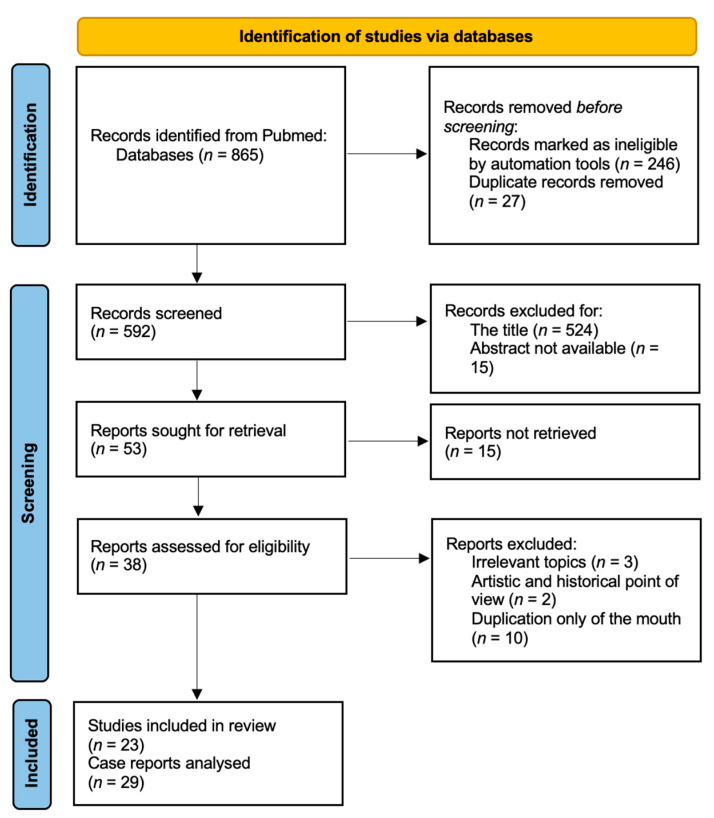
Data extraction method: In the PRISMA 2020 flow diagram for new systematic reviews, which included searches of databases and registers, the flow of information through the different phases of the systematic review is shown. It maps out the number of records identified, included, and excluded and the reasons for exclusions.

In total, 865 Pubmed CENTRAL studies were independently assessed by 2 investigators (LG and VT) using the PRISMA 2020 checklist (File S2) and explained in the flow diagram (Figure 1) [18]. We excluded all the studies with insufficient information, possible biases, contradictions, or inconsistent or arbitrary conclusions. We finally analyzed 23 papers corresponding to all the criteria selected; in these, 29 cases of diprosopus are presented. We extracted information about parental, gestational, clinical, genetic, and prognostic characteristics of individuals. The information that was extracted from each article is shown in Table 1 and Table S1.

## 3. Clinical Report

Our patient is the second-born daughter of healthy, Ethiopian, non-consanguineous parents. The mother was 22 years old (G2P2), and the couple had an older and healthy son. During the pregnancy, the mother did not take any drugs or “traditional medication”. The pregnancy was reported to be uncomplicated, but no blood screening tests or ultrasound scans had been performed.

At the 38th week of gestation, a female newborn was born by an assisted vacuum delivery in an Ethiopian hospital after a PROM (preterm rupture of the membranes) with an inadequate intrapartum antibiotic prophylaxis. The Apgar score was 4 at 1 min and 7 at 5 min of life, and the baby developed a mild respiratory distress syndrome that required intranasal oxygen administration for 12 h.

**Figure 2:**
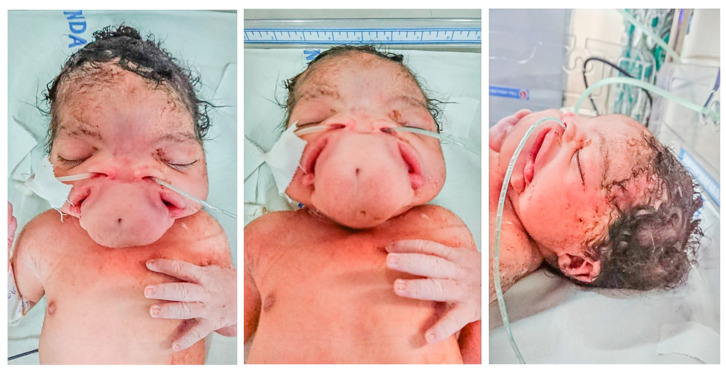
Photographs of the face of the proband showing the peculiar phenotype.

Her auxological data at birth were the following: weight 2800 g (18th centile; −0.91 DS), length 48 cm (27th centile; −0.6 DS), and head circumference 35 cm (84th centile; 0.98 DS). But what was striking was her facial phenotype. She presented widely spaced eyes, a flat nose without a tip, widely spaced nostrils, two oral cavities, two palates, two tongues, and a median protuberance with two pits between the two mouths (Figure 2); objective examinations were, otherwise, normal as well as the female external genitalia.

**Figure 3:**
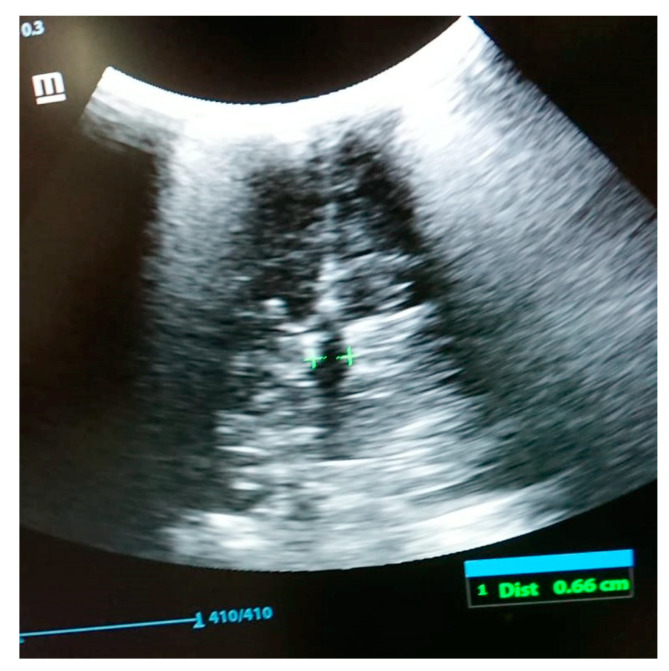
Brain ultrasound: enlargement of the third ventricle and agenesis of the corpus callosum.

**Figure 4:**
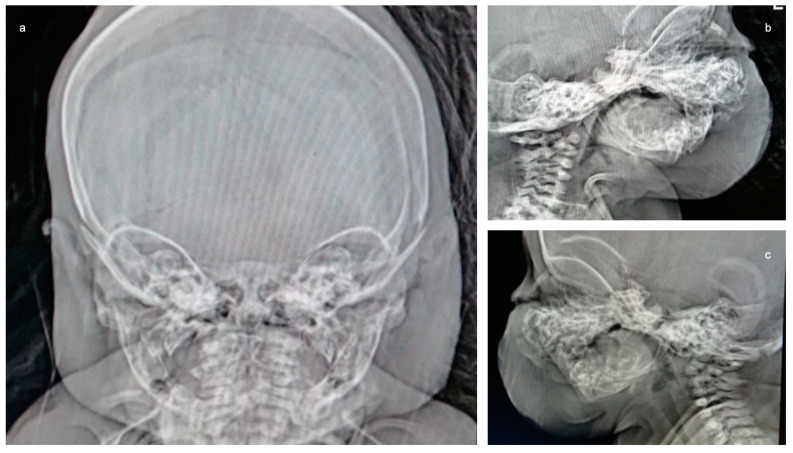
X-ray of the skull: On both the AP image and the lateral projections, it is difficult to recognize the duplication of the maxilla and mandible. The coronal and sagittal sutures are wide.

**Figure 5:**
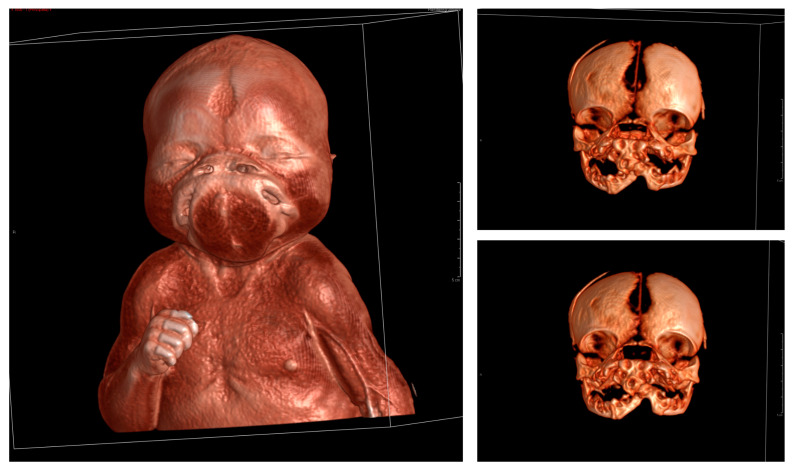
Cranial CT (3D volume rendering reconstruction): duplication of the mandible and, partially, of the maxilla, with partial duplication of the oral cavity and two distinct mouths. Note also the wide cranial sutures.

**Figure 6:**
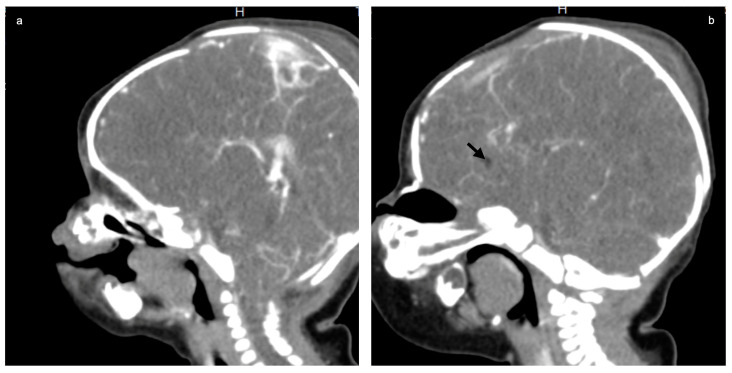
Brain contrast CT (sagittal MPR): agenesis of the corpus callosum (**a**) with a small anterior midline lipoma (**b**); black allow and small posterior fossa with cerebellar tonsillar ectopia, the tonsils extending below the level of the foramen magnum (**a**).

The following tests were carried out: brain ultrasound, skull X-ray, and head and neck CT scan. The brain ultrasound showed an enlargement of the third ventricle, and the corpus callosum was not detectable; since the examination was performed with an inappropriate probe (only the adult convex probe was available), it was not possible to visualize the cerebral cortex (Figure 3). On the skull X-ray, complete disorganization of the axillary and mandibular bones was revealed (Figure 4). To understand the malformation more precisely, a cranial CT scan was performed, which clarified the picture of bony disorganization of the maxillofacial complex; this examination revealed the presence of duplication of the mandible and, partially, the maxillary bones and the presence of a double opening of the oral cavity (Figure 5). Through CT image analysis of the central nervous system, it was possible to confirm the agenesis of the corpus callosum and the presence of a small anterior midline lipoma, a small posterior cranial fossa with the extension of the tonsils beyond the level of the foramen magnum was also found (Chiari I malformation) (Figure 6).

To complete the diagnosis, blood tests (WBC9 × 103/microL, RBC 4.62 × 106/microL, Hb 15.5 g/dL, HCT 42.7%, MCV 92.4 fl, MCH 33.5 pg, MCHC 36.3 g/dL, PLT 118 × 103/microL, Lym 27.5%, Neu 59.5%) and an abdominal ultrasound were performed on the third day of life and were found to be normal.

In conclusion, this is a case of diprosopus with associated brain malformations.

While we were studying the indication for a brain MRI to plan surgery, the child died suddenly in her crib at age 1 month. No autopsy was carried out, and unfortunately, no samples were preserved for genetic analysis. 

## 4. Results

The medical literature reports almost 40 cases of diprosopus up to now, but only 29 of them are fully analyzable in the literature (Table 1).

Analyzing patients described in the literature and our case, the mean maternal age is 27.4 years, and only in one case is the age over 40 years (data available in 25/30). During the pregnancies, no drugs or other substances recognized as teratogenic had ever been used.

Almost all cases present in the literature died within the first few months of life, and voluntary termination of pregnancy (VTP) was performed in six cases.

The average gestational age of the cases, considering VTPs, is 29.2 weeks, and the average birth weight is 1923 g (±285 g).

The prevalence of this condition is slightly higher in females (57%).

All the patients present the typical facial phenotype appearance. In particular, all subjects have two complete oral cavities, but only 50% have complete facial duplication consisting of the presence of two mouths, two noses, and four eyes.

Almost all the cases (96%) have different brain malformations associated with the characteristic facial phenotype. Anencephaly is the most common, present in 42% of the cases, followed by craniorachischisis, brain duplication, agenesis of the corpus callosum, and encephalocele. On the other hand, duplication of only the frontal lobes, thalami, cerebellum or brainstem, ventricular dilatations, hydrocephalus, and exencephaly are very rare. Agenesis of the corpus callosum is described in four reports [15,19,20], while in only two of these reports [19,20], there is a duplication of the frontal lobes or part of the brain.

Rare associated malformations are often found in other midline organs, and following brain anomalies in frequency are cardiac malformations (86%). These have a wide spectrum of presentation and range from complex cardiopathies, such as transposition of the great vessels, to trivial pictures characterized by interatrial defects (DSA). Less frequent associated anomalies include cleft lip/palate (63%) and diaphragmatic hernia (13%). 

Regarding the presence of genital malformations, it was difficult to assess their true incidence because they were described only in slightly more than half of the cases, and among these, two had ambiguous genitalia. The presence of duplications of other midline organs is sporadic, such as thymus, airway, or gastrointestinal tracts; the presence of omphalocele, alterations of the hepatobiliary system (absence of the gallbladder or hepatic dislocation), limb defects (club feet), or pulmonary hypoplasia is anecdotal.

Few genetic investigations have been performed in these patients. Karyotype was performed in only eight of the cases described in the literature, and it appeared to be normal. In one case, singleton WES was performed and did not reveal any causative variants.

## 5. Discussion

We present a case of a patient affected by diprosopus, with a clinical picture that falls within the most severe form described in the classification of Gorlin et al. [1]. To our knowledge, diprosopus (from the Greek διπρόσωπος, “two-faced,” from δι-, di-, hence two or double and πρόσωπον, prósopon, face) or diprosopia or craniofacial duplication is a very rare congenital disorder, in which the fetus is born with two faces. In the most pronounced forms, the newborn has complete duplication of facial features, and thus four eyes, two noses, and two mouths, but only one brain. 

The etiology remains unknown. The incidence of this malformation is <1/1,000,000, and the sex ratio is M:F = 1:1.4.

Diprosopus should be considered in differential diagnosis with other conditions affecting facial bones and midface abnormalities, in particular, supernumerary nostrils, medial cleft face syndrome, amniotic band syndrome, and lateral facial cleft. Supernumerary nostrils are not considered a duplication of the nose, as they are located above rather than between normal nostrils, do not form septa, and appear to be secondary to nasal pits [1,19,20,21,22,23,24,25,26,27,28,29,30,31,32,33,34]. Median cleft face syndrome consists of separation of the face in the midline without duplication of parts; it is thought to be secondary to a failure of neural crest cell migration over the frontonasal process. Amniotic band syndrome is secondary to an in utero traumatic event, including compression-related defects [35], typically resulting in clefts or disruptions of the face, as well as of the extremities and/or trunk [36]. Lateral facial clefting with maxillary duplication is considered a distinct entity [37].

As we see in the Results section, the mean maternal age is 27.4 years, and only in one case is the age over 40 years; only one case has a history of parental consanguinity, and often the couples had previous healthy children. 

In the most recent and followed-up pregnancies, when the diagnosis of diprosopus could be performed during the prenatal period, it always led to VTP. In addition, three cases of stillborn infants are described, and most of the subjects died within the first few hours of life and almost all within the first few months of life.

Confirming what is already known in the literature, prevalence is higher in the female sex.

As the classification drafted by Gorlin et al. [2] addressed facial phenotype appearance, all subjects have two complete oral cavities, but only 50% have complete facial duplication consisting of the presence of two mouths, two noses, and four eyes.

We emphasize that as many as 96% of these cases also have brain malformations. In particular, focusing on agenesis of the corpus callosum, it is described in only three other reports [15,19,20] besides ours; however, none of these present a midline lipoma and a Chiari I malformation.

Few genetic investigations have been performed in the reported patients, and none of them revealed a genetic cause; unfortunately, due to a lack of resources available in Ethiopia and due to the premature death of the child, genetic analysis could not be performed in our case. To better understand the genetic contribution to this disorder, it would be necessary to perform broad-spectrum DNA analysis in large cohorts of individuals, comparing their sequencing data, with a particular focus on the Shh pathway. The detection of novel genes must also be taken into account.

### Limitation of the Study

The lack of genetic data is an evident limitation of the description of the clinical case. In addition, case reports in the literature are not always complete, both from a clinical and genetic point of view. 

## 6. Conclusions

In conclusion, diprosopus is a rare congenital malformation characterized by complete duplication of the mouth or of the entire face. In almost all cases, malformations of the central nervous system are associated, and the most common is anencephaly.

The prognosis of this condition is usually unfavorable. Therefore, it remains essential to perform routine prenatal investigations, even in physiologic pregnancies, to recognize these conditions early and differentiate them from other conditions affecting the midface and facial bones to recognize the future possibility of intervention to correct the defect.

The etiology and pathophysiology of diprosopus are still unknown, and to date, no genetic alterations have been associated with this condition. The embryonic development of the head involves a highly complex and delicate process driven by a wide range of genetic components. The almost obligatory presence of cerebral malformations in diprosopus suggests a very disruptive event in the cephalic pole of the developing embryo. It could be interesting to study the condition in question both from an embryological and genetic point of view.

## Figures and Tables

**Table 1:** General information of patients described in the literature with diprosopus.

Clinical Data	Average
Maternal age (years)	27.4
Gestational age (weeks)	29.2
Weight (g)	1923
	**Incidence (%)**
Sex	57
Cerebral malformation	96
Anencephaly	42
Encephalocele	12
Cranial schisis- or rachischisis	35
Cerebral duplication	19
Agenesis of the corpus callosum	15
Facial malformation	100
Cardiac malformation	86
Cleft lip/cleft palate	63
Diaphragmatic hernia	42
DSD	13

## Data Availability

The data that support the findings of this study are available from the corresponding author upon reasonable request.

## Supplementary Materials

The following supporting information can be downloaded at https://www.mdpi.com/article/10.3390/genes14091745/s1. File S1: CARE13 checklist. File S2: PRISMA 2020 checklist. Table S1: Phenotype and clinical history of patients described in the literature with diprosopus.
